# Reimagining consumer involvement: Resilient system indicators in the COVID‐19 pandemic response in New South Wales, Australia

**DOI:** 10.1111/hex.13556

**Published:** 2022-07-04

**Authors:** Patti Shih, Laila Hallam, Robyn Clay‐Williams, Stacy M. Carter, Anthony Brown

**Affiliations:** ^1^ Australian Centre for Health Engagement Evidence and Values (ACHEEV), School of Health and Society University of Wollongong Wollongong New South Wales Australia; ^2^ Sydney Local Health District Sydney New South Wales Australia; ^3^ Centre for Disability Research and Policy (CDRP) The University of Sydney Sydney New South Wales Australia; ^4^ Centre for Healthcare Resilience and Implementation Science (CHRIS), Australian Institute of Health Innovation Macquarie University Sydney New South Wales Australia; ^5^ Health Consumers Sydney New South Wales Australia

**Keywords:** consumer engagement, consumer participation, consumer partnership, COVID‐19 pandemic, health system resilience, Panarchy, patient and public involvement

## Abstract

**Background:**

Reflections on the response to the COVID‐19 pandemic often evoke the concept of ‘resilience’ to describe the way health systems adjusted and adapted their functions to withstand the disturbance of a crisis, and in some cases, improve and transform in its wake. Drawing from this, this study focuses on the role of consumer representatives in healthcare services in initiating changes to the way they participated in the pandemic response in the state of New South Wales in Australia.

**Methods:**

In‐depth interviews were conducted with two cohorts of consumer representatives. Cohort A included experienced and self‐identified consumer leaders, who worked together in a *COVID‐19 Consumer Leaders Taskforce*; Cohort B included participants outside of this group, and purposively included consumer representatives from rural and regional areas, and culturally and linguistically diverse communities.

**Results:**

The pause in consumer engagement to support health service decision‐making in responding to the pandemic forced consumer representatives to consider alternative approaches to participate. Some initiated networking with each other, forming new collaborations to produce consumer‐led research and guidelines on pandemic‐related patient care. Others mobilized support from community and politicians to lobby for specific healthcare issues in their local areas.

**Conclusion:**

The response to the COVID‐19 pandemic made visible the brittle nature of previous engagement processes of involving consumers in organizational design and governance. However, the momentum for proactive self‐organization in an unexpected crisis created space for consumer representatives to reset and reimagine their role as active partners in health services. Their ability to adapt and adjust ways of working are key assets for a resilient health system.

**Patient or Public Contribution:**

This project is a collaborative study between academic researchers and health consumer (patient and public) representatives. It followed the principles of codesign and coresearch, whereby both consumer representatives and academic researchers contributed equally to all stages of the project. The study was cofunded by both academic institutions and consumer representative organizations.

## 1 INTRODUCTION

### 1.1 Resilience of health systems in a pandemic

Reflections on COVID‐19 pandemic responses often evoke the concept of ‘resilience’ to describe how health systems were challenged by, yet withstood, a resource‐intensive and rapidly evolving crisis.^1^, ^2^, ^3^ Health systems are ‘complex adaptive systems’ made up of both humans (e.g., clinicians, patients, consumer representatives, administrative staff, policy makers) and nonhuman components (e.g., hospital structures and resources, clinical care processes, governance policies).^4^, ^5^ Their capacity to be responsive and make constant adjustments before, during or following both expected and unexpected events are key to resilience in these systems.^6^ Indeed, studies show that health systems with components that were agile, adaptive and encouraged flexibility tended to do better in the pandemic, while more rigid systems were forced to change.^1^, ^2^, ^7^ In Australia, healthcare organizations made rapid changes for better functioning during the pandemic, including introducing new policy advisory groups,^8^ reforms in patient care protocols,^9^ reallocation and co‐ordination of roles and resources between public and private hospitals and adoption of telehealth and its inclusion as a Medicare (public funding) billing item. Some argue that these changes would have otherwise ‘taken decades’.^10^

Resilience is related to a theory from ecology known as ‘Panarchy’, where cycles of growth and destruction occur alongside changes in system connectedness. Resilience is depicted as a core dimension in this adaptive cycle.^11^ The theory of Panarchy proposes that unexpected catastrophes can break bonds and reduce connectedness within a system, releasing enormous amounts of energy that is then available for rapid reorganization and new growth.^12^ In this respect, a crisis such as a pandemic may also present an opportunity, in the form of a valuable point of reflexivity and transformation, that can help improve the quality and functions of a health system.^8^, ^13^

This article focuses on the potential for adaptivity and resilience among members of the community who work within health services as representatives of health consumers. Health consumers (see Box 1) are patients, families, carers and members of the community who are current, previous or potential users of health services.^14^

BOX 1 **Terminology used to describe different types of consumer interactions**

*Health consumer*: A person who has used, or may potentially use, health services, or is a carer for a patient using health services.^14^ Consumers include patients, families, carers, friends and other support people.^15^ The term was first adopted by people who used mental health services in the 1990s and emerged from a rights‐based discourse with a focus on the concept of ‘consumer rights’. In more recent years, the term has become contested, and it has been criticised, by some, for being part of a market‐based discourse. It is used in this article to reflect its usage in most contemporary Australian health policy.

*Consumer representative*: A health consumer who has taken up a specific role to provide advice on behalf of consumers, with the ultimate aim of improving healthcare.^16^ They provide a consumer perspective, contribute consumer experiences, advocate for the interests of current and potential health service users and take part in decision‐making processes.^14^ A consumer representative is committed to representing not just their own perspectives or experiences; their input is often informed by feedback and the views of other consumers.^14^

*Consumer engagement*: The process of involving consumers^17^ in the planning, design, delivery, measurement and evaluation of systems and services.^14^ It is usually initiated by the health organisation or system.

*Consumer involvement*: Any activity that involves consumers, regardless of quality or depth.

*Consumer participation*: When consumers, carers and community members are *meaningfully involved* in decision‐making about health policy and planning, care and treatment, and the well‐being of themselves and their community.^17^ It differs from consumer involvement in that it is the activity that is considered to be *meaningful by the consumer involved*, in addition to the organization, and participation differs from consumer engagement in that it is the *meaningful contribution* from the consumer following the process of engagement.

*Partnering with consumers*: Partnering is the principle and commitment to share each aspect of the decision‐making (including the development of alternatives and the identification of the preferred solution)^18^ from planning, design, delivery, measurement and evaluation of systems and services.^14^

Consumers are a crucial source of healthcare knowledge and solutions. Their participation in health services decision‐making as citizens and end‐users of healthcare contributes to the political legitimacy of these processes.^19^, ^20^ Moreover, their input in health services and health research in both routine healthcare^21^, ^22^, ^23^ and in crisis situations such as pandemics and natural disasters is considered crucial in improving design, quality and innovation.^24^, ^25^ However, the international literature suggests that the participation of consumers and the broader community in COVID‐19 responses was very limited. In the urgent rush to respond to outbreaks, healthcare services tended to rely on biomedical and bureaucrat‐controlled approaches with limited community involvement.^26^, ^27^, ^28^

Despite this, innovative and community‐ and consumer‐led responses to the pandemic emerged to support government and clinically led initiatives.^29^, ^30^ Informed by the framework of health systems resilience and the theory of Panarchy, this study examines changes in how consumers participated in healthcare service design and delivery in New South Wales (NSW), the most populous state in Australia, during the pandemic.

### 1.2 Consumers participating as ‘partners’ in health services

**Figure 1:**
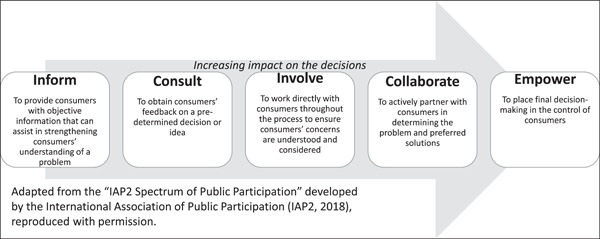
Spectrum of health consumers' engagement.

In Australia, the National Safety and Quality Health Service (NSQHS) Standards, developed by the Australian Commission on Safety and Quality in Health Care, views consumers as ‘partners’ in healthcare services.^14^ The Standards require organizations to create and demonstrate systems and strategies supporting involving consumers in the development and design of quality healthcare, in addition to ensuring that patients are included as partners in their own care. ‘Partnering’ with consumers stipulates their active, effective and impactful participation in priority‐setting, design, delivery and evaluation of health services and systems. Rather than resting solely on traditional forms of informing and consulting consumers, participation entails meaningful involvement and active collaboration. The International Association of Public Participation developed a well‐known spectrum to demonstrate the potential range of involvement levels, from minimal to full consumer involvement, which we received permission to adapt in Figure 1.^18^ The further to the right of the spectrum an activity is, the deeper the level of participation,^31^ and the stronger the consumers' role in decision‐making.^32^ As Gill and Gill^33^ suggest, ‘professionals must relinquish control as consumers accept greater responsibility’.

The participation of consumers in health services is a mandated NSQHS accreditation standard. At the service and system‐design levels, this includes the role of consumer representatives, whose input is described by the NSQHS as informed by their own experiences as well as by the feedback of other consumers.^15^ Health system stakeholders in NSW have made considerable investments to engage consumers including by employing consumer and community engagement managers, although strategies can vary.^34^ Health consumer peak bodies, such as the Consumer Health Forum of Australia (CHF) and state‐based Health Consumers New South Wales (Health Consumers NSW) also facilitate consumers participating in the health system, by supporting their training, recruitment and advocacy. The underlying goal is that consumer representatives become embedded into the decision‐making processes of the health system, rather than acting as ‘outsiders’ who are informed and consulted before or after the fact. Nevertheless, research shows inconsistent levels of meaningful participation across and within different organizations, sectors, disease groups and socioeconomic communities, and challenges in achieving effective partnership in a complex political, social and operational environment.^32^, ^35^, ^36^, ^37^ Individual consumer representatives also differ in their ability to influence decisions, often depending on their experience and level of support from host organizations; organizations also vary in their capacity and capability to organize and support consumers.^35^, ^38^

The direct and indirect impacts of the COVID‐19 pandemic on patient care services and health outcomes in NSW have been reported by several authors,^2^, ^39^ including some reporting concerns raised by consumer representatives.^40^, ^41^ Dimopoulos‐Bick and Walsh^40^ suggested that avenues of consumer representation were severely reduced in the early period of the pandemic outbreak. Besides that study, limited research has been conducted regarding the impact of the pandemic on consumer participation in the Australian healthcare system.

This project emerged from frequent informal feedback about novel consumer representative activities during the pandemic to Health Consumers NSW, a peak body for health consumers. These initial informal interactions led to a collaboration with academic researchers to systematically examine and theorize the role of consumer involvement in responses to the pandemic in the NSW health systems and services. The aim of the study was to (1) collect diverse consumer representatives' accounts of engaging with health services in NSW during the COVID‐19 response in 2020; (2) systematically examine drivers of consumer involvement and advocacy in relation to the COVID‐19 pandemic; and (3) analyse these events from the perspective of systems resilience. The analysis focuses on experiences of the first period of the pandemic outbreak, which culminated in a state‐wide lockdown from March to July in 2020, and was before vaccine availability. Data collection concluded in mid‐2021, shortly before NSW experienced the significant outbreak of the COVID‐19 Delta variant, coupled with a slow start to the vaccine rollout.

## 2 METHODS

### 2.1 Design

This is a qualitative in‐depth interview study with health consumer representatives in NSW. They are typically members of the community who volunteer their time to represent patients, carers and the community at large in health service committees, across hospitals, Local Health District (LHD) boards, primary health networks and nongovernment health organizations. As a collaborative study between academic researchers and consumer representatives, it followed the principles of codesign and coresearch.^21^, ^42^, ^43^ The project was cofunded and collaboratively developed; data were coanalysed by researchers and consumer representatives. The study involved the guidance of external advisers from government organization representatives from the NSW Agency of Clinical Innovation and NSW Ministry of Health.

### 2.2 Sampling and participant recruitment

Recruitment and sampling occurred in two phases. In the first phase, we purposively sampled 14 experienced consumer representatives from across NSW who were members of the COVID‐19 Consumer Leaders Taskforce (hereafter referred to as ‘Leaders Taskforce’), a health consumer network formed online during COVID‐19 (Cohort A). An email invitation was sent to the group mailing list of the Leaders Taskforce by Health Consumers NSW; members actively opted in by contacting the University of Wollongong research team, and Health Consumers NSW did not know who had agreed to participate. Data collection took place between October and December 2020. After discussing these preliminary data with the study team and the external advisors, it was decided that further recruitment was needed to understand the perspectives of other consumer representatives not involved in the Leaders Taskforce, and balance the sample. An additional cohort was selected to include more rural, regional and culturally and linguistically diverse (CALD) participants, and with varying levels of experience as consumer representatives. This additional group (Cohort B) was recruited and interviewed between April and June 2021. An email was sent to consumer participation managers of local hospitals and LHDs to pass onto their consumer representatives, who then expressed their interest in taking part in the study by contacting the University of Wollongong research team.

Respondents were eligible to participate if they were active consumer representatives for at least 6 months before the NSW COVID‐19 lockdowns in March 2020. For both cohorts, potential participants completed a brief screening questionnaire. This part of the study provided participants with (1) study information and (2) an opportunity to express interest in taking part in the interviews. The screening questionnaire collected data on (1) basic demography and (2) brief information about participants' role in health services in NSW, and any changes experienced during COVID‐19. These responses guided the purposive participant selection in Cohort B, which aimed to reflect diversity of geographic locations, gender balance and CALD inclusion. In addition, it allowed for specific interview questions to be appropriately tailored. For Cohort A, the first 14 respondents to respond to the survey were recruited. For Cohort B, there were 39 eligible respondents. Sixteen of these were purposively selected to maximize participant demographic diversity.

### 2.3 Data collection and analysis

One‐on‐one interviews were conducted via telephone or a video conferencing program by P. S. We avoided face‐to‐face interviewing because of the need to socially distance during the pandemic, and remote interviewing enabled more effective engagement of participants from a diversity of geographic locations across NSW. The interviews averaged 45 min (ranging from 33 min to 1 h 15 min) and focused on the individual consumer representative's experience before and during the COVID‐19 pandemic. Interviews were audio‐recorded and professionally transcribed. Names of participants and any other information that would lead to the identification of participants (e.g., geographic locations, names of individuals and organizations) were deidentified to protect participant privacy and confidentiality.

The research team included L. H. (a consumer‐researcher and member of the Leaders Taskforce, who was not a study participant), A. B. (the Executive Director of HCNSW and Honorary Research Fellow at UOW), P. S. (UOW health sociologist), R. C. W. (Macquarie University researcher, resilience in health systems expert) and S. C. (UOW public health and health services researcher). Each team member had different roles in analysis. First, the transcribed and deidentified textual data from Cohort A were imported into NVivo qualitative analysis software, and analysed by P. S. to derive a set of broad themes and subcategories. Cohort B was then recruited and interviewed, and the data were transcribed and deidentified, then coded by P. S. using the same broad set of themes. To protect the identity of participants, L. H., as a member of the Leaders Taskforce, accessed only deidentified data extracts from Cohort A, already organized under the broad themes by P. S. L. H. accessed complete deidentified transcripts from Cohort B. P. S. and L. H. met regularly to discuss data analysis, and regularly fed their analysis back to A. B. and S. C. for input. R. C. W. provided further coding and conceptual advice around the theoretical concept of systems resilience and Panarchy.

## 3 RESULTS

A total of 30 participants were recruited (Table 1). The majority of Cohort A (*n* = 14) participants had more than 7 years of experience as consumer representatives. More than half resided in metropolitan locations, with the rest being from regional and rural areas. In Cohort B (*n* = 16), five participants had 4–6 years' experience, and eight had been consumer representatives for 1–3 years: one participant had been a consumer representative for only 6 months before the COVID pandemic. Participants varied in terms of the areas that they focused on (e.g., mental health, aged care, children's health), and the level of decision‐making that they were involved in. Four participants from Cohort B specifically identified as consumer representatives of CALD communities, providing insightful information about CALD experiences (see Section 3.3). In total, nine participants came from one LHD, four each from three LHDs, three from one LHD, two from another LHD and one each from four LHDs; only three LHDs were not represented.

**Table 1:** Participant demographics

		Cohort A (*n* = 14)	Cohort B (*n* = 16)	Total (*n* = 30)
Gender				
Female		10	10	20
Male		4	6	10
Residential geographic type^a^				
Metropolitan		8	4	12
Regional		5	5	10
Rural		1	7	8
Years active as health consumer representative				
<1		0	1	1
1–3		4	7	11
4–6		1	5	6
7+		9	3	12

^a^ ‘Regional’ is a sociogeographical definition used in Australia to describe populated regions outside of the major metropolitan areas.

We present the results in four sections: We consider how consumers perceived and understood the reasons for, and implications of, pausing consumer participation during the pandemic; consumers' experiences of forming new self‐organized networks or political allies; their connections or disconnections in the wider community; and finally, the perceived short‐ and long‐term outcomes and lessons learned from their experiences during the pandemic. Illustrative quotes attributed to individual participants organized in subthemes are marked with deidentified participant codes, with a letter beginning ‘A’ or ‘B’ referring to their allocated cohort.

### 3.1 Theme 1: Health organizations pause engaging consumers during the pandemic response

A common way for consumer representatives to describe the overall health service response to the COVID‐19 pandemic was ‘command and control’. The urgent focus on acute hospital care and outbreak control demanded a top‐down chain of command. This was a necessary and pragmatic approach in a crisis situation. The priority of pandemic‐specific service design paused almost all regular, business‐as‐usual service design and improvement work with consumers and clinicians alike (see the illustrative quotes in Table 2, subtheme 1.1).

**Table 2:** Theme 1 illustrative quotes: Pausing consumer engagement

Subtheme	Exemplar
1.1. Understanding the need for a ‘command‐and‐control’ approach to the pandemic	Everything else has been deprioritised because COVID is taking all the focus and energy within the system. So it's not that [consumers] were no longer important and what was being worked on is no longer relevant; it's just everything has had to be sidelined for a while. But I think unfortunately the precedent for including consumers is not solidly established. I think that's the summary statement about that. I think they have a long way to go to know how to involve consumers even though they have designed the terms of reference where they say we are going to be involved, and that they will come and talk to us, or ask us to come and talk to them, but it didn't happen. (A02) A lot of things were done without consumer involvement from the get‐go. You know, consumers being amiss, ‘nothing about us, without us’, and consumer engagement really has to begin like this. Because of the intensity of the pandemic and the dramas with which various government departments were having to deal with on a daily basis, and having to come up to speed themselves, they didn't have the capacity to deal with being inclusive of consumers right at the beginning. That is seen as a sort of, I suppose it's a breach of faith by many people. But I suppose we also have to be tolerant of the fact that it was something new and far more dramatic than anyone expected it to be. Being pragmatic about it, that was almost understandable, but not entirely forgivable. (A10)
1.2. Varied levels of health service commitment to maintaining consumer participation determined at the individual leadership level	There was such strong commitment in our LHD, that we had to keep pressing on. Because I was one of the people who questioned whether we should have put the board consumer committee on hold for one or two meetings [due to COVID lockdowns]. The Chief Executive Officer and the Director of Clinical Governance and the board chair, and I, all discussed it, and in the end we decided, no, we don't want to do that. We're just getting momentum here and okay, we don't have the staff at the moment, but there are things we can still do. (A04) The Director of Nursing was not truly committed to consumers engagement], [they] were just convening the meetings because they had to. It has a very low priority. I think COVID is just an excuse. (B06)
1.3. Impact of pausing consumer participation and the active participation of consumer representatives	I suppose because you're dealing with bureaucrats, they then miss out on the wealth of experience consumers can bring. Because consumers are the foot soldiers, the people out there who are in the communities, involved, interacting on a daily basis with the health system, and people with complex health needs. They [the health service] miss out on some of the things that are important, and perhaps be more easily solved if they caught them right at the beginning. (A10) I think it's the fall back position for the Ministry of Health. They really think that consumer engagement really takes time. It's nice to have but not essential. These are quite old‐fashioned views, really, that still, kind of, pervade the whole system. When things get tough, people fall back on that older way of thinking. (A09) For a couple of months we had nothing, and once we resumed our online meetings, you would get reports from people within the hierarchy about what was happening in the hospital but it was always in the context of ‘this is what we are doing and we're letting you know’. It wasn't ‘this is what we've decided to do about the hospital visitation policy during COVID, what do you think?’, or ‘this is what we've done to cancel all these clinics, what do you think will be the impact on consumers’ or, ‘do you have concern about that as a consumer representative’? (A11)
1.4. Impact on patient care	I feel that we are part of the quality improvement process. And if we're not there, then they're not getting the feedback that they need about what it's like to be a consumer of their health services. (B06) Before COVID, I suggested a program for mental health. Mental health for my [CALD] community, to get educated with this program. Because of COVID they stopped the plan, and stopped the consumer input into that plan. So even when I give my input, it didn't get implemented quickly. After COVID it start to move again. But during COVID mental health was really very important. (B01)

Abbreviation: LHD, Local Health District.

Individual health services determined whether and how to engage with consumers, resulting in most halted their consumer engagement activities. While, for some consumer representatives, there was on‐going and regular communication of key pandemic directives from the Ministry of Health, LHD or the local hospital, these interactions tended to focus on information provision, rather than participating in the development, design or governance of services. Due to social distancing rules and non‐essential travel, and the fact that some consumer representatives were from populations more vulnerable to COVID‐19, most health services postponed face‐to‐face committee meetings indefinitely, although a few adapted to online conferencing quickly to maintain routine consumer involvement. Initially, recognizing the uncertainty and the intense strain that health services staff and clinicians were under, and to avoid any further burden, consumer representatives broadly adopted the approach of stepping back to ‘let clinicians and staff do their job’. However, as months passed and the bulk of consumers engaged in supporting services remained paused, significant policy and service design decisions were made without consumer representatives. A key example is the management of family visitations in healthcare facilities during the early period of the pandemic in 2020, which was left to the discretion of each LHD or facility. Most organizations, in their attempts to manage infection control, classified patient visitors as an infection risk and placed blanket bans on nonpatients entering facilities. While some visits were allowed by exception, in most cases, the exclusion of family presence to support presenting patients actually created additional staff burden. Where family members would usually be there to attend to the physical and emotional needs of patients, this was now left to staff. These early visitation policies resulted in poorer patient experiences, and was likely to have directly impacted on patient outcomes. Whereas staff perceived families as infection control risks, patients needed family members to support them. Without family visitors, backgrounding patient information, which is crucial for supporting accurate diagnosis, was less comprehensive; discharge home was less informed and co‐ordinated; and patients suffered poorer mental and emotional health.

Of the few examples where consumers were involved in pandemic‐related service design, low‐level effectiveness was reported. For example, one study participant (B14) was invited to participate in reviewing an amended Emergency Department protocol, but the highly technical nature of the discussions and language excluded the consumer representative from contributing in a meaningful way. Another participant from a rural community (B12) was asked by the health service to convene a ‘Pandemic Risk Committee’ that consisted of first responders such as the police, ambulance, fire departments and the State Emergency Services. They convened the committee, but were excluded from attending. Another consumer representative raised concerns about a CALD community being singled out with abuse and described as ‘vectors of the virus’. They asked the local health service to respond to dispel the myths and support the community. Their formal letters to the hospital hierarchy were not responded to or acknowledged (B10).

There was an expectation among consumer representatives that they would eventually be reactivated to contribute to the pandemic response, alongside clinicians and first responders. As their exclusion over time continued, consumer representatives began to question their fundamental role in health service design. Experienced consumer representatives, for example, lamented the almost overnight reversal of decades of improvement of consumer participation, recounting how it had ‘reverted to the old ways’ of doing things. One participant outlined their frustration, noting that the pandemic appeared to be ‘an excuse’ to not involve consumers (B06). The exclusion was also described as a ‘breach of faith’ of the consumer partnership mantra of ‘nothing about us, without us’ (A10) (see also Table 2, subtheme 1.3).

One of the most pressing concerns for consumers about their absence in the pandemic response was the potentially avoidable impact on patient care as health services rapidly adopted new ways of working (see other examples in Table 2, subtheme 1.4). Consumer representatives were also increasingly concerned about the cascading effects of cancelled or delayed clinics on patients' physical and psychological well‐being and health outcomes, particularly for people newly diagnosed or living with life‐threatening, life‐limiting or chronic conditions.

### 3.2 Theme 2: Forming new consumer networks and research

In the absence of regular consumer representation channels to and interactions with health services, alternative means of contributing patient perspectives were sought. In light of the feedback from its membership, Health Consumers NSW called out for a specific group of experienced consumer representatives to lead new ways of engaging during the pandemic in April 2020, shortly after the first state‐wide lockdowns began. This consumer‐led group, ‘Leaders Taskforce’ (from which Cohort A participants were drawn), actively sought out the views of health consumers in their state to inform system and service responses to the pandemic. One way was via ‘Amplify’, a health consumers online forum, inviting and supporting other consumers not in the Leaders Taskforce to contribute feedback for research.^44^ The group also organized a number of national consumer workshops, inviting participants to share their own experiences and views, and collected consumer feedback from Care Opinion Australia. Over the course of 2020, the Leaders Taskforce conducted their own research and developed position papers from the perspective of patients and consumers. The first focused on patient visitation in health facilities, and the importance of, and guidelines for, supporting family presence.^45^ The second focused on telehealth.^46^

This collaboration required consumer representatives to adapt quickly to online forms of networking, and working *with each other*. Until then, most consumer representatives were unknown and unconnected to each other. Predominantly, they had attended face‐to‐face meetings in health services, often operating alone and disconnected from each other. According to Cohort A participants, the clear agenda for the Leaders Taskforce to identify and address pandemic‐specific concerns from a consumer perspective, grounded in research evidence that the group had generated themselves, was key to the effectiveness and impact of the collaboration. Some suggest that this was an important reflexive exercise, producing new insights as well as new self‐assurance to take ownership of self‐produced research evidence (A02; see also other examples in Table 3). Apart from the research led by the Leaders Taskforce, Cohort A participants also took part in other consumer‐led research activities among pre‐existing consumer structures. This included feedback, surveys and workshops conducted by several patient groups including CHF, the national consumer organization.

**Table 3:** Theme 2 illustrative quotes: Networked consumer groups

Subtheme	Exemplar
2.1. Formation of new consumer networks builds confidence and proactivity among consumer representatives	People within the Leaders Taskforce spoke of the difficulty of getting access back to the committees that they were previously doing. Consumers, I think, as a result of COVID, have networked between themselves much more. The Leaders Taskforce would not have happened before [the pandemic], and being able to get to know all those connections. The opportunities to address concerns and do the position statement within [consumers' networks] propelled us further to collaborate. (A06) The Leaders Taskforce gave some evidence and consolidated or focused voice around particular issues, which then gives us information that we can speak with more confidence to when we're engaging within the system, in terms of the weighting of that evidence and also the confidence around that material. Because we can see that a group of people have actually contributed to that conversation or position statement or policy statement, and we're very clear about what that actually means for us as health consumers within the system (A02) Some consumers have become, and maybe I'm one of them, more aware of becoming more proactive and certainly a lot more of my consumer interests are around research and about how consumers need to become involved in developing research. (A11)

### 3.3 Theme 3: Connections and disconnections within the wider community

When recruiting Cohort B, we purposively included consumer representatives outside of the Leaders Taskforce, to compare and examine other consumer‐led activities during the pandemic. Consumers in this cohort experienced similar challenges of being excluded from health services decision‐making, and also identified inadequate and inappropriate aspects of patient care during the pandemic. Accounts from consumer representatives in rural and regional areas reaffirmed issues that existed before the pandemic, including human resource shortages. Although rural and regional communities experienced fewer COVID‐19 outbreaks in 2020 compared to metropolitan areas, preparing communities for the pandemic nevertheless exacerbated these resource limitations.

Participants in Cohort B tended to already be active members of their own local communities. During the pandemic, many were themselves involved as volunteers in community organization‐led initiatives to support socially isolated older people or provide financial assistance where government‐led services were overstretched or absent. Without the expected input into local health services, some Cohort B consumers proactively leveraged different avenues of political support, from their communities, politicians or different levels of the health system to address concerns during the pandemic.

For consumer representatives in rural and regional NSW, the area of concern and therefore impact of their work were more specific to their locality or community compared to the broader state‐wide agendas of the Leaders Taskforce. For example, a midwifery‐mother's group in a regional NSW area was concerned about the well‐being of birthing mothers restricted to having only one person accompany them at birth. They were successful in lobbying for visitation rules to be changed via their connections with the LHD, and over‐riding Ministry directives. This change was specific to one local hospital and one patient group, in contrast to the broader system‐wide policy approach of the Leaders Taskforce.

In a small rural township, consumer representatives at the local hospital objected to a proposed COVID‐19 testing site creating a disruptive and unsafe location for traffic and public safety. The decision had been made by hospital executives without consumer input, and their objections were initially dismissed. Concerned consumer representatives then leveraged the support of the LHD (one administration level above the local hospital), who organized a ‘fly‐in’ squad of logistical managers to evaluate the testing site. Recognizing the problems, they recommended a change to a safer location as suggested by consumer representatives (B12). In another rural township, where health services staffing was already in shortage, the Visiting Medical Officer (VMO), the only accessible doctor, became unavailable due to pandemic travel restrictions, and was subsequently disengaged without consultation with consumer representatives. The delayed process of replacing the VMO propelled consumer representatives to organize a public community townhall meeting, drawing media and political attention to the issue, resulting in the prompt resolution to the crisis (B11).

New collaborations were formed with community networks outside of health services and with nonconsumer representatives to highlight the needs of the community and to address them. Participant B13 suggested that the local health services committee that they served was neither effective nor transparent in addressing identified consumer and community needs during the pandemic. Dissatisfied with traditional consumer participation opportunities set by bureaucratic structures, they set up a health action group with the support of local politicians, with the aim of achieving greater and more meaningful community impact (see Table 4, subtheme 3.3).

**Table 4:** Theme 3 illustrative quotes: Local consumer‐led activities

Subtheme	Exemplar
3.1. Locally specific networks were successful in lobbying for locally specific or condition‐specific healthcare issues	The Midwifery [group] [in regional town] did get [hospital visitation policy] changed earlier in the piece, because they were concerned about the birthing mother not being able to have any one there, they're only allowed one person. They really lobbied hard, and they did get [the visitation policy at the local hospital] changed. So even though the health providers were saying that they had to abide by the government directives, but they were able to get changes for birthing mothers. (B02)
3.2. Leveraging political support from the local community, media and access to politicians	[The Visiting Medical Officer (VMO) could not service the local hospital during COVID, and was not replaced]. So, it got to the point where I then said, right, I'm going to call a town meeting … I was told we could only have 110 people and 450 turned up. I advertised it via email, text and social media. The idea was to let the community know that we don't have full‐time VMOs and do you want to form a sub‐committee, a community town committee and we will fight to get doctors. If we can get more GPs, it's less pressure on the hospitals. But the media – everyone turned up, [member of parliament] came – so, that pushed – and at the moment we have full‐time VMOs. (B11)
3.3. Forming political allies and networks with a less bureaucratic structure outside of healthcare services committees	So there's a danger that managers and district chief executives are more concerned – and in some ways they're bound to in terms of their service agreements – to be consummate public servants rather than actually engaging with the real needs of the communities. So some of us [worked] together with some of the local councillors to form a [health action group]. I have been working with another group in town where we are looking at developing [a community health forum] and we will probably do that in conjunction with our [health action group]. It will meet every two months and its aim will be to get all stakeholders members of the community, doctors, allied health people, any businesses to come together to define what we see as the real needs of this local community and then to try and develop a relationship with the Ministry for Health as one that is around win/win rather than us going cap in hand to them. (B13)
3.4. Disconnections in CALD communities due to the pandemic	For us consumer representatives in [a CALD community], what make it more difficult is the inability for us to reach the greater number of people in our community. Many people do not actually know that this role exists and what it does, they don't have this information. And even if so, the reluctance of some of them to meet us because of their concerns about COVID. And because of COVID, the number of people using the health facilities has decreased. So they don't have any ways of raising issues about the health services. (B07)

Abbreviation: CALD, culturally and linguistically diverse.

However, it should be noted that not all participants from Cohort B were involved in consumer‐led pandemic responses. Some did not identify the need to do so, while others did not have the opportunity, capacity or community connections or support from elected officials. A consumer representative from a CALD community, for example, said that they felt disconnected from their community during the pandemic. Due to what they saw as inadequate culturally and linguistically appropriate information and education, the widespread fear of infection stopped some members of their community from reaching out to consumer representatives, while others were simply unaware that consumer representatives existed as a community resource (B07). Similarly, for another CALD translator and consumer representative, replacing face‐to‐face translation services with telephone‐based translation meant that their service was far less effective because they were unable to build rapport and engage with their clients prior to and following the formal clinical consultation. It was during these pre‐ and postmeeting discussions that they noted that older CALD patients often socially connected, and would raise personally significant issues, such as loneliness and financial stress. The consumer representative's effort to reinstate face‐to‐face translation services in their local hospital following lockdown was unsuccessful, with hospital administration citing financial constraints (B03; see also Table 4, subtheme 3.4).

### 3.4 Theme 4: Learning and outcomes from consumer‐led pandemic responses

The pandemic was an unexpected ‘shock’ that reset new ways of thinking and doing things. In reflecting on the many self‐initiated consumer‐led activities that evolved during the pandemic, consumer representatives described the pressures and disturbances of a crisis as an unexpected opportunity, a ‘circuit breaker’ (A13) that forced them to be creative in rethinking and resetting their ways of operating. The pandemic also created a need for them to proactively interact with health services and the broader health system if they were to be effective representatives of the people and communities who used and depended on health services. As one participant suggested, consumer participation during COVID was sustained *outside* of health services *by consumers themselves* (A06) (see further illustrative quotes in Table 5, subtheme 4.1).

**Table 5:** Theme 4 illustrative quotes: Learning and outcomes from consumer‐led pandemic responses

Subtheme	Exemplar
4.1. The pandemic was an unexpected ‘shock’ that reset new ways of thinking and doing things	[Before the pandemic] people were not thinking outside the square and there probably weren't enough consumers saying, ‘oi, just a minute’, so it took a circuit breaker like COVID. (A13) I think the health system has probably improved, because they're not taking for granted that everything will always be easy. They will always be alert to the fact that all of a sudden they could have to transition from one mode of operation to another, very quickly. (B04) I think [the pandemic] has very much brought to the attention on how the VMO system worked. Why have we got no VMOs? The obvious answer would be because of COVID. But it wasn't that great before. We just relied on [the LHD] because that's their job. And what's happening with our dialysis machine? What's going on with the Aboriginal Health Service? Why aren't we talking with them? All the nursing homes, why aren't we all more connected? I think that COVID has brought the community together. You can't do it by yourself. You have to take a group of people with you. (B11) [COVID‐19] encouraged innovation and creativity and doing things differently. And it does stretch traditional models of healthcare a lot, and what can be delivered in different ways. So, certainly, things in our organisation which we've never been able to do before, we are able to do because of COVID. And so it has been a really good enabler in that way because it just warranted such an urgent response. (B05)
4.2. The wider impact of consumer‐led research in improving healthcare and strengthening the health system	Every LHD has a different policy for visiting. There is no state policy. When the [Visitation paper by the Leaders Taskforce] was distributed, that was picked up very, very quickly by the LHDs, by politicians as well. (A06) You're discovering that we can come together as a group electronically without ever meeting, provide a lot of input to position papers on hospital visitation went up to the Ministry. We got invited to present to a hospital Emergency Department. A couple of the Leadership Team talked to them about what consumers were saying about it, which came through [*Consumer peak body online forum*]. We got a lot of feedback from consumers through *[online forum]*. So, it's all a brave new world, it's operating in a different way. I think from a consumer's perspective, there will be different expectations. That's quite positive. (A13) What I find really interesting, though, which has become more evident during COVID, from my perspective, is how a lot of the work that Consumer organisations are involved in around shared decision‐making, social prescribing, all those sorts of things, is being considered, worked on, and advocated by clinical groups as well. And there's a coming together. I know of an individual practitioner was actively working within her [clinical specialty] and she embedded shared decision‐making with her patient cohort. So as well as the peak bodies becoming much more visible within the whole area of consumer engagement, we've also got local individual clinicians picking up on the importance and value of consumers knowledge (A07)

Abbreviations: LHD, Local Health District; VMO, Visiting Medical Officer.

There were wider impacts of consumer‐led research in improving healthcare and strengthening the health system. An unintended outcome was that the experience heightened consumer representatives' confidence and expectations to become central and proactive partners in the health system. Networking with new allies, whether with other fellow consumer representatives or more broadly with their local community, meant that they were more likely to be better informed, and could contribute more confidently, effectively and to more healthcare issues in their individual consumer representative roles. The consumer‐driven research outputs developed by the Leaders Taskforce and other consumer groups also began to fill an information void, by identifying and consolidating consumers' needs and perspectives to inform health services design during the pandemic. These were reportedly well received by state and local health services as well as politicians (see further illustrative quotes in Table 5, subtheme 4.2).

The publications released by Health Consumers NSW and the Leaders Taskforce focused specifically on the needs of patients and families as they navigated their healthcare needs during the pandemic. This was an attempt to balance the prevailing public narrative about the impact of the pandemic on staff alone. The publications were sent to the Chief Executive and Clinical Governance Executive of every LHD and health pillar organizations in the state, as well as executives in the state Ministry of Health. In turn, some staff supported and forwarded the documents broadly within their organizations. In September 2020, the landscape began to shift on visitation rights following a focus by the Clinical Excellence Commission NSW on releasing updated guidance on supporting visitation while mitigating and minimizing infection risks.^47^ The “Visiting Guide: Family Presence in a COVID‐19 Normal World” by the Consumer Leaders Taskforce^45^ released in August 2020, specifically outlined the need for families to be present in support of hospitalised patients, while still in co‐existence and supportive of broader infection risk mitigation strategies. The documents continue to be used and referenced by current NSW Health initiatives. Most recently, the contribution of the publications and its authors was recognized by the NSW Minister for Health in a recent March 2022 briefing on family presence and visitation.

## 4 DISCUSSION

The policy commitment to partnering with consumers is reflected in the strong presence of consumer representatives throughout health services in NSW well before the COVID‐19 pandemic. Yet, the participation of consumers in the pandemic response at the start was noticeably absent as the health system largely paused interactions with consumer representatives. In facing an unexpected healthcare crisis, the intuitive reaction was to default to a traditional command‐and‐control and biomedically centred healthcare response, which also excluded ‘non‐essential’ work such as consumer representation, and in turn the perspective of their patients in the system and service decisions made.^28^ Even at the highest level of consumer participation impact (see Figure 1), the bureaucratic system of health service organizations remain the drivers of decision‐making by determining how consumers will be involved. Where active consumer participation was maintained, it was due to individual leaders or ‘champions’ in health services, rather than a systemic trend. This ‘traditional’ hierarchy of consumer participation, or what Ehlrich et al.^48^ describe as ‘rules of engagement’, is reflected by the fact that consumer representatives were willing to step back and allow health services and clinician groups to respond to the crisis at its peak. Consumer representatives were willing to occupy the lower end of the participation spectrum during an exceptional crisis when it was perceived to be a short‐term emergency response. However, continued exclusion as the pandemic progressed impacted the trust that consumer representatives had in the system and their place in it, with impact on patient outcomes.^25^, ^40^, ^49^

From a resilience theory perspective, a system without adequate participation of consumers was not robust enough to withstand the unexpected turbulence of a crisis. The *brittleness* of previous connections within health services was therefore prone to being broken.^1^ Yet, in a remarkable demonstration of proactive self‐organization, consumer representatives showed that they were capable of developing alternative approaches to effectively bring patient and community voices into pandemic‐impacted service design and delivery.^50^ This was driven by the monumental shift in how the system was operating in a healthcare crisis that left a vacuum in place of previously structured, albeit inconsistently, consumer participation. Illustrating the Panarchy phenomena, the pandemic was a fluctuating and turbulent event that loosened previous interconnectedness between consumers and the healthcare system and ‘released’ the energy and momentum for consumer representatives to rapidly adjust and reorganize alternative forms of influence.^12^ In the absence of developed structures or plans to involve and integrate consumer representation in a pandemic of the scale of COVID‐19, there was room for consumers to drive and determine the shape of their input in pandemic‐related issues. The result was that the pandemic triggered a key transformation of innovative consumer participation.

The opportunity to resort and reorganize ways of working in the health system created new opportunities for consumers to initiate and trial alternative ways to participate. A number of different tactics were identified among the two consumer cohorts recruited in the study, who were purposively sampled to explore the range of consumer experiences and responses during the pandemic. There were two significant differences between the two groups: first, the *level of experience*, and second, in terms of *interconnectedness*: Cohort A was comprised predominantly of experienced and seasoned consumer representatives, who had convened and networked during 2020 as the pandemic first began to surge; Cohort B was made up of consumers with varying levels of experience, who were not operating as a network connected to each other, but instead as individual consumer representatives within their local areas. A key point of difference with networked consumer representatives in Cohort A is that developing consumer‐driven research in COVID‐19 presented a point of consolidation, collaboration and legitimization of their work. Their COVID‐19 position papers concretely demonstrated the legitimacy and importance of consumers' knowledge, skills and lived experience to represent their communities and support responses to the pandemic. For such networks to continue and flourish after the pandemic, consumer peak bodies play a key role in supporting collaborative and inclusive research activities. For Cohort A consumer representatives, online collaborations were necessary for complying with social distancing regulations, but also encouraged and enabled engagement with a broader and more diverse spectrum of health consumers outside of their pre‐existing networks. Participants in Cohort B also demonstrated quick adjustment to the way they participated in healthcare service design, although their approaches tended to involve the broader local community, political allies and the media outside of health services. The effectiveness and accessibility of more traditional forms of activism available perhaps reflect the tight‐knit nature of regional and rural communities.

As online engagement and video conferencing become more normalized forms of communication as a result of the COVID‐19 pandemic, the digital space can be better harnessed to bring together previously dispersed consumer representatives from diverse geographic areas, or flexibility for those who are less able to meet in person due to lack of resources, time commitment or a disability. This is more conducive to finding new political allies to advance consumers' voices outside of traditional avenues. This also highlights the crucial role of Health Consumers NSW as a consumer peak body in providing resources to support and facilitate this new work. There are, however, deeper systemic issues about resource allocation, managerial will and support for CALD communities in particular that will remain prominent. These are not only real‐life challenges during and after the pandemic but also signal limitations of the system in tailoring support CALD and other less represented communities. As shown in the more severe Delta outbreaks in 2021 in NSW, it was rural, lower socioeconomic‐status and CALD communities that suffered most adverse effects, which is due in part to the very health service inadequacies identified in this study.

As both partners in the health system as well as representatives of the wider community, consumer representatives have the distinct characteristic of being both ‘inside’ and ‘outside’ of the health system. They are ‘outsiders’, representing the perspectives of patient, family and community stakeholders who are outside of the day‐to‐day running of the health system. They are also ‘insiders’ when invited to participate with staff in health service planning, design, delivery and measurement. The fluidity between the two is unique and crucial to connect the voices of the community (‘outside’) to the health system (‘inside’). This study demonstrated the ability of consumer representatives to make links with each other and their communities via self‐organization. At the same time, consumer representatives had developed a strong baseline of trust from decision‐makers. For example, the trust attached to the individual consumer representatives helped achieve acceptance of the research outputs of the Leaders Taskforce, increasing the willingness of healthcare staff to take on board the findings more broadly.

However, this means that as a group, consumer representatives have to self‐manage their positions to retain that trust. It requires representatives to be tactful and careful with the use of language and being positioned ‘in the court’ of decision‐makers, yet also speak ‘the truth’ as outsiders that often insiders (such as healthcare service staff members) cannot. There are also recognizably other risks to the new ways of working among consumer representatives. While it is often easier to initiate and unite individuals who have shared problems and disenchantment, it may be more challenging to maintain the network through a process of developing and navigating a shared vision of goals. Nevertheless identifying shared goals is key to whether the group can maintain bonds and momentum over time once more traditional consumer representative roles re‐emerge. However, it is anticipated that the benefits have outweighed the risks. The knowledge gain afforded to consumer representatives who are better networked with each other and their patient bases will also improve their individual patient and community stakeholder advocacy contribution to service design and delivery. It also will potentially improve their standing as effective contributors with their staff peers, and as a contributing network, will improve their credibility and legitimacy in uniting recognized and experienced voices.

### 4.1 Study strengths and limitations

This study uses health systems resilience theory to interpret shifts in the consumer participation landscape during the first year of the COVID‐19 pandemic in NSW, providing a fresh approach to understand how consumers have the power to enact change. The empirical elements of the study were conducted in one state in Australia only; however, the conceptual observations can be applied in other settings, as similar circumstances have also been observed internationally.^28^ Our study participants were not intended to be generalizable sample of all consumer representatives in NSW and their views may not reflect the experience of other consumer organizations or networks. A limitation of our sample is that one LHD was strongly represented in the data, while three were not represented. This may have more strongly influenced the analysis. While participants' background and interests are broad‐ranging, participants were purposively selected from a larger group who self‐selected. Although the Leaders Taskforce was a specific group that may not be representative of a full range of consumer and healthcare experiences, it was geographically diverse when it was convened. In sampling for Cohort B, we prioritized demographic diversity. The two cohorts were interviewed at different times (6 months apart) and as the COVID‐19 pandemic as well as political engagements were shifting. Their views may therefore be informed by the time‐specific context of their experience. The longer time lapse between the start of the pandemic and when Cohort B participants were interviewed potentially gave this group more time to reflect on how their experiences have shaped how they have adjusted their now‐returned routine work as consumer representatives.

## 5 CONCLUSION

Before the COVID‐19 pandemic, reforming health systems, including integrating meaningful consumer participation, was relatively slow moving.^13^ The unexpected crisis presented an unintentional but timely opportunity to consolidate and focus energy for consumer representatives working in the health system. It created space for consumer representatives to reset and reimagine their role as active partners in health services. As it is characteristic of human agents in a complex adaptive systems to reorganize and adapt to improve the resilience of the system, consumer representatives have shown, in their ability to do so, and doing it well, that they are a crucial asset for a resilient health system. In this respect, consumer representatives were the ones that shifted the partnership from the level of ‘inform’ and ‘consult’ to ‘empower’ during the pandemic (see Figure 1). ‘Empowerment’, in this context, was in the ability of the consumer representatives to defy the prevailing absence of their involvement in the system and service and to initiate alternative and novel ways to bring the voices of patients, families and communities together and into service design and decision‐making.

It is likely that this new way of working is better adapted to a postpandemic healthcare system and likely to be more robust to future perturbations. As suggested by Kleefstra and Leistikow,^28^ involving consumers in the postpandemic world should become a ‘new normal’, rather than an afterthought. The success of some consumers in finding new, and potentially more effective, ways of working suggests potential future models for this ‘new normal’. Consequently, their success and effectiveness must be evaluated from angles outside of traditional health service‐oriented frames.

The focus now, as the initial shock of the pandemic subsides, should be to embed the innovations that have been shown to be effective and strengthen the new interconnectedness between consumers, and between consumers and system and service providers as the new adaptive cycle begins. Planning and policy for future emergencies should explicitly include the use of new and existing infrastructure for genuine and deep consumer participation. It is also important to remember the lessons into the future as this is unlikely to be the last shock that the health system suffers. Rather than clinging to the status quo, decision‐makers should recognize and leverage opportunities for system improvement when they arise as a consequence of unanticipated events.

## Data Availability

The deidentified data that support the findings of this study are available on request from the corresponding author. The data are not publicly available due to privacy or ethical restrictions.
